# “Honey, Milk and Bile”: a social history of Hillbrow, 1894–2016

**DOI:** 10.1186/s12889-017-4345-1

**Published:** 2017-07-04

**Authors:** Jonathan Stadler, Charles Dugmore

**Affiliations:** 1 Wits Reproductive Health and HIV Institute (WRHI), Faculty of Health Sciences, University of Witwatersrand, Johannesburg, South Africa; 2 Witwatersrand University History Workshop, 1 Jan Smuts Avenue, Braamfontein, Johannesburg, 2000 South Africa

**Keywords:** South Africa, Hillbrow, Health, Social History

## Abstract

This commentary constructs a social history of Hillbrow, an inner-city suburb in Johannesburg, South Africa, based on a review of relevant published historical, anthropological and sociological texts. We highlight the significant continuities in the social structure of the suburb, despite the radical transformations that have occurred over the last 120 years.

Originally envisaged as a healthy residential area, distinct from the industrial activity of early Johannesburg, Hillbrow was a prime location for health infrastructure to serve the city. By the late 1960s, the suburb had been transformed by the rapid construction of high rise office and apartment buildings, providing temporary low cost accommodation for young people, migrants and immigrants. In the 1980s, Hillbrow defied the apartheid state policy of racial separation of residential areas, and earned the reputation of a liberated zone of tolerance and inclusion. By the 1990s, affected by inner-city decay and the collapse of services for many apartment buildings, the suburb became associated with crime, sex work, and ungovernability. More recently, the revitalisation of the Hillbrow Health Precinct has created a more optimistic narrative of the suburb as a site for research and interventions that has the potential to have a positive impact on the health of its residents.

The concentration of innovative public health interventions in Hillbrow today, particularly in the high quality health services and multidisciplinary research of the Hillbrow Health Precinct, creates the possibility for renewal of this troubled inner-city suburb.

Positioned within the economic capital of South Africa, present-day Hillbrow serves as a kind of ‘sanctuary’ that presents economic opportunities for poor, displaced, and marginalised people from within and outside South Africa. It is a place of strangers in which it is possible to shelter and forge a new life, a place of “flow, of entry and exit”, of hopefulness and opportunity [1]. Yet, it is also an uncertain “fragmented space” of “poverty, violence and dysfunction that emerges directly from apartheid and global inequalities” [1]. Our title for this commentary, borrowed from Phaswane Mpe’s acclaimed novel, captures the paradoxes and contradictions of everyday life in Hillbrow. He writes, “…welcome to our Hillbrow of milk, honey and bile, all brewing in the depths of our collective consciousness…” [2].

As the articles in this series attest, Johannesburg’s inner-city suburb of Hillbrow and its surrounds provide an exemplary setting for exploring the complex processes of urbanisation and how this shapes the health of its residents, and in particular, the spread of and response to HIV. With a conservative estimate of 75,000 people living in approximately 200 buildings, some over 15 stories high, spread over approximately one square kilometre, Hillbrow is one of the densest urban areas in southern Africa (68,418 per km^2^). Nearly 10,000 apartments, a handful of hotels and freestanding houses accommodate 24,857 households [3].

Once a “bohemian”, fairly “wild”, neighbourhood [4], in which counter-cultural identities flourished, it is now regarded as “dirty, diseased and dangerous”, a space dominated by drugs, sex work, crime and grime [5]. This transformation in its social character is routinely portrayed as a sharp break between the past and the present. Indeed, Hillbrow residents who, in the vast majority, are black^1^ and relatively recent arrivals, have little to connect themselves to neighbourhood histories prior to the 1990s [6]. Meanwhile, former residents of the suburb, for the most part white, lament these changes as personal losses of a ‘golden era’^2^.

However, an overview of the social history of Hillbrow reveals a picture of enduring continuities across its 120-odd years of existence. For most of its history, Hillbrow has served as a port-of-entry for migrants and immigrants, and nurtured a highly transient population. It has also been a space in which informal and illegal economic activities are used by residents to survive. In other respects, Hillbrow can be regarded as a space of social change, host to pioneering political, social and cultural transformations. Emerging from the epicentre of capitalist investment in the mining industry in the late nineteenth century, and later offering a solution for low cost accommodation, it was one of the first urban areas to challenge the apartheid state’s racist residential policies through concerted civic action; it was the focal point for organised gay and lesbian activism; and finally, it was the site of the first hospital services in Johannesburg. These events mark Hillbrow as distinctive in the context of South African urban spaces and even within histories of the post-colonial city more broadly. Accordingly, Hillbrow’s long history of political and social temerity and its receptiveness to cutting-edge ideas suggest that it is also a space in which innovative responses to the HIV epidemic in this region may find a home.

We begin our overview by locating Hillbrow within the broader context of the intensive industrialisation of Johannesburg at the turn of the twentieth Century, and then proceed to explore the changes that have taken place over the next hundred years. Our particular focus is on Hillbrow’s rapid growth, shifting demographics, social character, and early responses to the AIDS epidemic.

## Setting the context: early Johannesburg, 1880–1950

The formation of Johannesburg followed the discovery of alluvial gold on the Rand (a ridge of gold-bearing rock) in the 1880s. By 1914, the South African mining industry was producing 40% of the global gold output, and economic policy had shifted from agricultural to industrial investment. Johannesburg emerged as the commercial and political centre of this transformation. Initially unused land for grazing and the occasional digger’s shack, the triangle of land called Randjeslaagte defined the borders of the city until 1902 [7]. By 1914, the mining camp of 3000 diggers had been transformed into a city of a quarter of a million residents [8]. Both white and black men were employed by the mines, and by the end of the 2nd World War, an estimated 100,000 unskilled black workers resided in mining compounds and rented accommodation in and around Johannesburg.

The 1930s to 1950s witnessed the arrival of significant numbers of black men and women from the Native Reserves^3^. In strictly utilitarian economic terms, they moved to Johannesburg to access the higher wages offered by the burgeoning manufacturing sector, having been driven away from the countryside by the erosion of the rural subsistence agricultural economy. While men sought employment, women in turn sought their husbands who had abandoned them in the Native Reserves [9].

The flow of black migrants into the city from the countryside resulted in a peculiar form of urbanisation. Although now resident in Johannesburg, men and women continued to regard the countryside as home, preferring to move between the urban and rural settings. A close inspection of migrant lives in this early period reveals that many sought to isolate themselves from assuming a permanent urban identity, a phenomenon demonstrated in part by the high rates of turnover in the labour force [9].

The demographic profile of settlements on the Rand fostered a specific gender distribution in migrant communities. While white men outnumbered white women two to one, this asymmetry was even more pronounced in black settlements. Within the city centre there were more than 10 men for every woman, while in the residential compounds the ratio increased to 24 to one [8]. The scarcity of women residing in the city promoted masculine cultures in which alcohol consumption and commercial sex featured prominently [8]. Liquor was initially used by employers as a means of controlling and pacifying the workforce [10], later becoming a major problem for the mining industry [11]. The pronounced gender imbalance meant that working men sought sexual relief and companionship amongst commercial sex workers. In 1895, there were an estimated 97 brothels in Johannesburg and one in every ten women over 15 years of age was a sex worker. Most were migrants from the Cape Colony and European countries following efforts to outlaw prostitution there. In Johannesburg, commercial sex followed the European model of intermediaries (pimps) controlling female sex workers [8]. The area between Bree Street in the north and Anderson Street in the south, Sauer Street in the west and Kruis Street in the east, contained most of the town’s brothels and became known as ‘Frenchfontein’.

By 1915, the police had succeeded in forcing many of the pimps and sex workers out of business [8]. However, a less visible form of sex work combined with beer brewing and selling continued to provide black migrant women with a source of income [12]. This informal economy was to become increasingly important as the city drained men from the rural areas, prompting large numbers of women to move to Johannesburg in their quest to gain autonomy from their rural households [9, 13].

Black women making homes for themselves in the backyards and shacks of vacant land in the city were seldom married. In the 1890s, only 2% of the 1678 black women in Johannesburg were classified as married. Very few were formally employed; occupations usually reserved for women in domestic service such as washing and housework were dominated by men [8]. As described in Ellen Hellman’s account of ‘Rooiyard’, which provides an intimate view of everyday life in early twentieth century Johannesburg, by the early 1930s an increasing number of black men and women migrants from the countryside lived in “yards” in what is now the inner-city of Johannesburg [14]. The yards were congested spaces; rooms were leaky and hot and lacked ventilation. Working men’s wages were usually inadequate to pay rent and support their families. A few black women supplemented household incomes through domestic work in White households, but most earned cash by brewing and selling beer (*skokiaan*). Although Hellman’s descriptions of yard life stress the urbanisation of African cultural practice, she notes the necessity of residents retaining links with their rural homes for rituals of marriage, birth and death [14].

The process of rapid urbanisation resulted in health problems: pneumonia, dysentery, tuberculosis (TB), typhoid, scurvy and other nutritional disorders were common. TB in particular was a major cause of morbidity and mortality amongst black labourers [15] and there were frequent outbreaks of smallpox, with two major epidemics (1893–94 and 1897–98). Dysentery and typhoid similarly took on epidemic proportions, largely a result of overcrowding and inadequate supplies of clean water. Pneumonia and respiratory illnesses were more prevalent in winter. Death from injury due to mining accidents was high; for example, early records reported that almost 6 out of 1000 miners died in a single year [11].

It is in this rapidly expanding, industrialising city – with burgeoning challenges in health and urban planning – that the suburb of Hillbrow was conceived.

## Hillbrow is born and grows: 1894–1978

*“The situation was found to be so charming, standing as it does high up, and in the cases of very many of the plots, with such grand views of the whole of the Golden City (…) Hillbrow was (…) going to become the fashionable residential suburb of Johannesburg.”* [16]

**Fig. 1:**
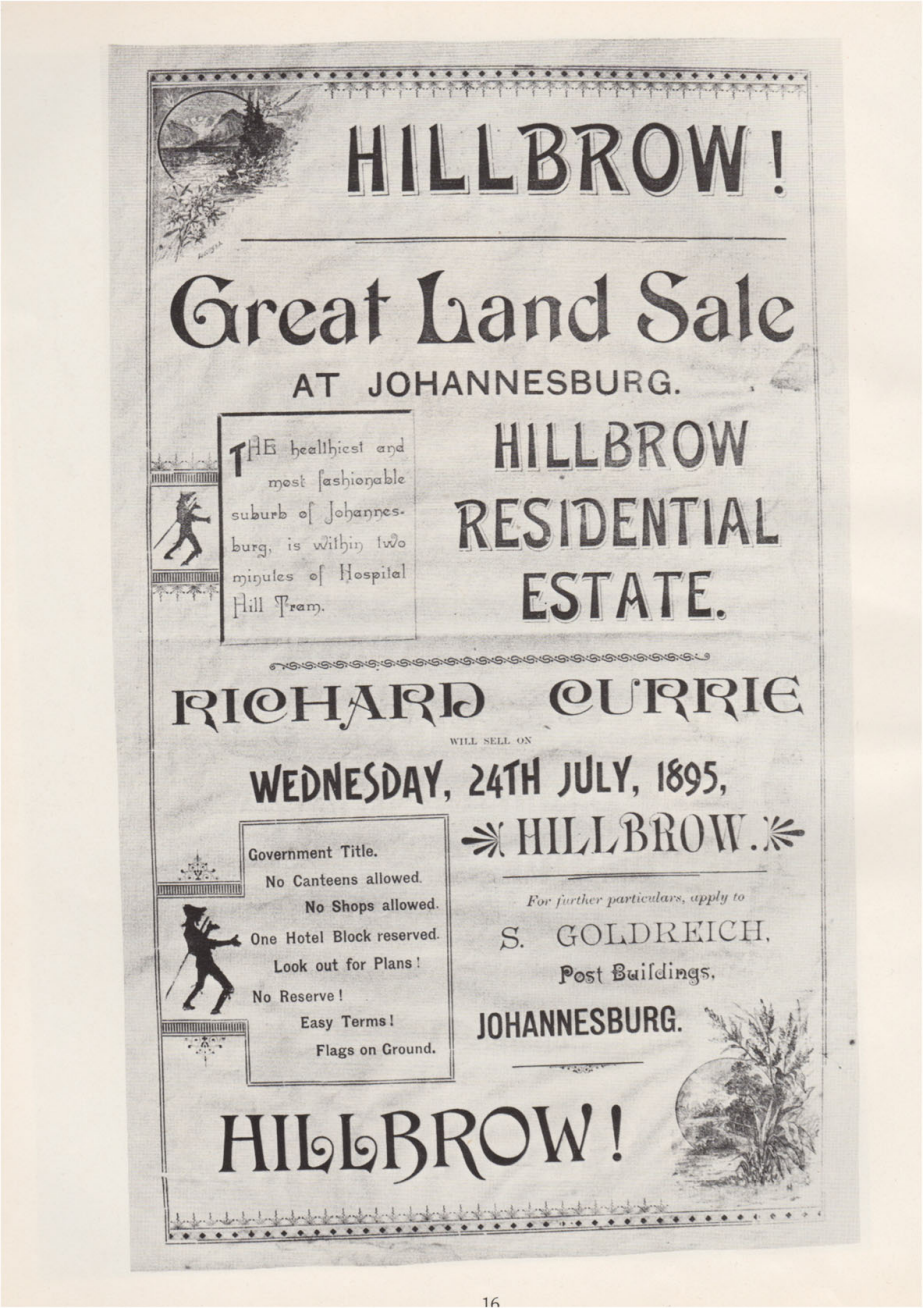
An announcement of land for sale in Hillbrow in 1895. Don Nelson Publishers kindly gave permission to publish this image [18]

**Fig. 2:**
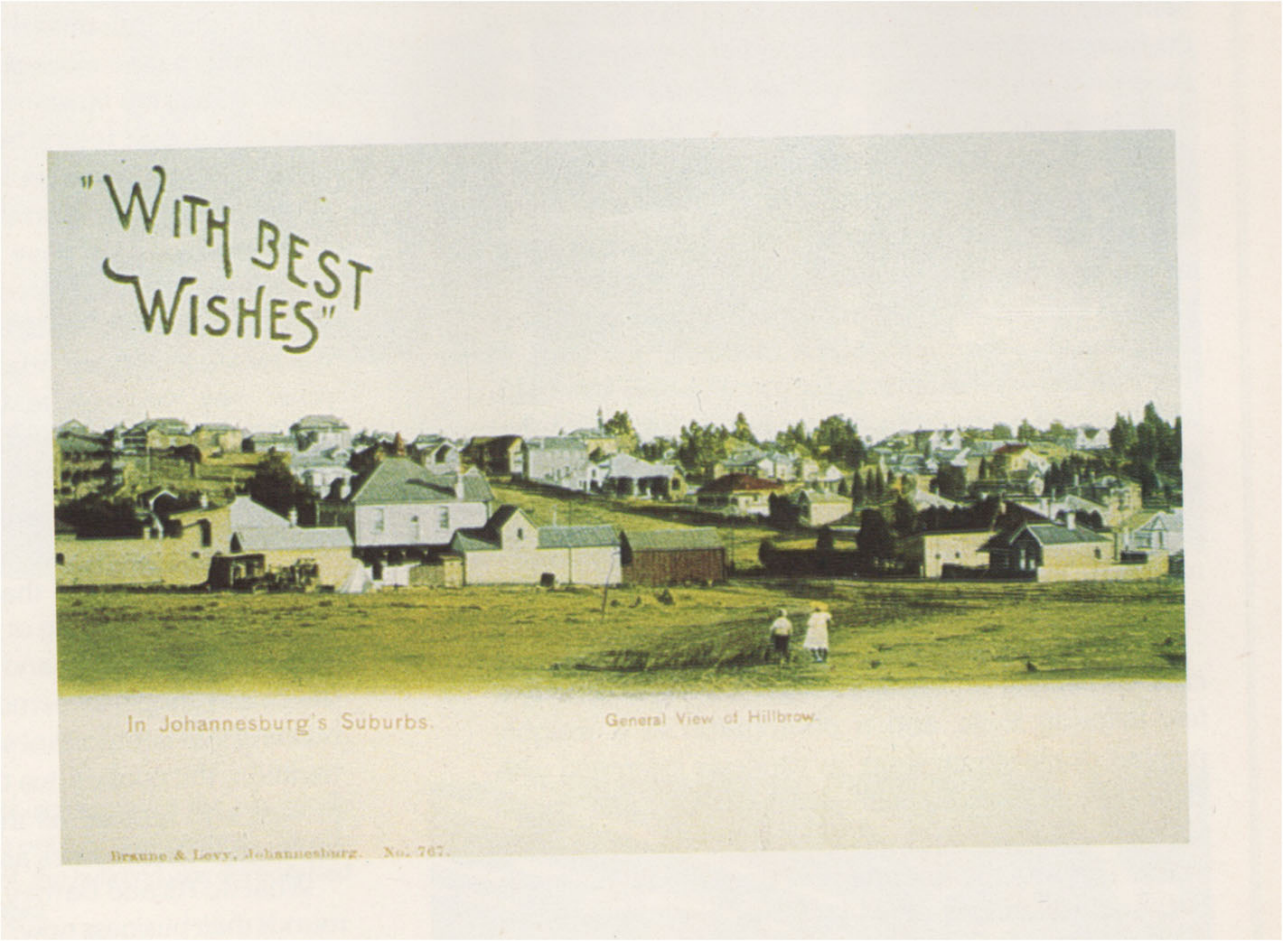
An early 1900s postcard depicting suburban Hillbrow. Don Nelson Publishers kindly gave permission to publish this image [18]

Located on the far northern section of Randjeslaagte, Hillbrow was established on the 24th July 1895 as an upscale residential area consisting only of detached houses. Neither shops nor ‘canteens’ (restaurants and taverns) were originally allowed in this ‘healthiest and most fashionable suburb of Johannesburg’ [4] (Figs. 1 and 2). In an echoing of Late Victorian and Early Edwardian ideas about the importance of clean air for health, Hillbrow – with its lofty position over the sprawl of diggers camps and the dusty city bowl – was considered the ideal location for the establishment of health services. Johannesburg’s first hospital was built in 1889, replacing the make-shift infirmary located in the city jail, and it housed the first operating theatre, built eight years later [7].

Hillbrow enjoyed two massive growth spurts in the twentieth century. By the 1920s, new technology (elevators and reinforced concrete) meant that it was possible to build a few multi-storey buildings, although height restrictions were enforced and private houses still far outnumbered apartment buildings by the early 1940s. With the removal of height restrictions in 1946, however, Hillbrow entered an intensive phase of construction that lasted until the late 1960s. Over this period the number of apartment buildings increased by 250%, while office space doubled. Hillbrow became a high density, high-rise, inner-city neighbourhood, only one kilometre from the centre of Johannesburg [4]. Building construction extended outwards towards the neighbouring suburbs of Berea and Yeoville. However, largely due to the implementation of the Rent Control Act of 1949, which was intended to protect the interests of white working class tenants [17], no new building developments occurred after the end of the 1960s.

In the early decades of the century, additional health facilities were developed to meet the growing need. Many of these were located on ‘Hospital Hill’, the region adjoining Hillbrow to its east. Here, a ‘non-European hospital’ was established in 1921, followed by a branch of the Johannesburg Hospital in 1924, and a nursing training college, based in three houses across the road. Also on ‘Hospital Hill’, the Transvaal Memorial Hospital, better known as the ‘Children’s Hospital’, was built in 1923.

By the 1940s, specialised maternity services emerged in the area. Queen Victoria Maternity Hospital (originally established in Siemert Road in central Johannesburg in 1913) was moved closer to Hillbrow in 1943, in its relocation in the neighbouring region of ‘Joubert Park’. In the 1950s, the Florence Nightingale Hospital (Maternity Hospital) was built in the same street as the Women’s Prison – now part of Constitution Hill. Indeed, this point overlooking Hillbrow housed other health institutions, including the Johannesburg Fever Hospital, established in 1916 behind the Civic Centre in Hoof Street, opposite the Women’s Prison.

Collectively, and with the establishing of later private and government hospitals such as the Rand Clinic in Bruce Street, the Brenthurst Clinic in Clarendon Place, and the Parklane Clinic just beyond, these health facilities contributed to the cementing of Hillbrow’s identity as a ‘health precinct’ by the 1960s. Yet at the same time, the suburb was gaining a reputation for sex, prostitution and disease.

This emerging reputation was shaped largely by the type of accommodation offered for rental in Hillbrow, and assumptions about its associated social character. A large number of the apartments were designed as single rooms – known as ‘bachelor flats’ – the “ideal fluid accommodation situation for transients” [18]. This feature contributed considerably to the suburb’s character as a place for immigrants and young people. From the 1960s, Hillbrow also attracted young white couples who moved out of their parents’ homes and lived in the relatively cheap apartments until they had saved sufficient money to buy a house in suburbs further from the city centre. South Africans moving to Johannesburg from elsewhere in the country also used Hillbrow as a base for a few months until they could buy or rent better accommodation, while businessmen from out of town stayed in Hillbrow’s many hotels when in Johannesburg for business. Perhaps the most striking feature of this period in Hillbrow, and most evident in ex-residents’ reminiscences of living there post-World War II, was its cultural and linguistic diversity. In the 1960s, approximately 30% of Hillbrow residents were foreign born, predominantly Europeans [4]. The continental flavour of Hillbrow was reflected in the abundance of foreign language bookshops, restaurants, cafés, bakeries and delis.

## Hillbrow becomes integrated: 1978–1990

*“Hillbrow was on the ‘cutting edge’ of apartheid’s social, political and geographical demise.”* [19]

Hillbrow’s population soon far outstripped the 40 per acre predicted in 1946 by local authorities. By the 1980s density had increased to 180 persons per acre (400 per hectare). The skyline was now dominated by the J.G. Strijdom Tower (Post Office Tower), built in 1971 and stretching 269-m high, forming the iconic image now instantly recognisable as a symbol of Johannesburg itself. But more than such physical developments, this period is marked by Hillbrow’s significant challenge to apartheid legislation governing where ‘non-whites’ were permitted to reside.

Hillbrow was designated as a ‘white’ area under the Group Areas Act^4^ that was promulgated in 1950. While over 1000 black residents lived in the suburb, largely working in local apartments and offices as cleaners and domestic servants, according to the 1970 census, they kept to themselves in small rooms on the rooftops of the buildings in what was dubbed by the media as ‘locations in the sky’ [20]. Meanwhile, the white population at this time numbered 10,000, with a mere handful of Indian and coloured residents. The overwhelming ‘white’ character of Hillbrow could be seen in its streets, as the Pass Laws^5^ meant that only those who worked or lived there could avoid arrest from ubiquitous police inspections.

The erosion of the Group Areas Act began in the period 1978 to 1982, as Indian and Coloured residents tentatively began to enter Hillbrow in greater numbers. In part, this movement was triggered by a serious shortage of accommodation in ‘Indian’ and ‘Coloured’ areas and later in black townships as well. The latter resulted in a steady trickle of black people seeking out flats in Hillbrow in the mid to late 1980s. Many were also trying to flee the violent clashes between ANC-aligned youths and hostel-dwellers that had consumed the townships by this time [21].

These accommodation shortages in other parts of the city coincided with a surplus of housing in Hillbrow, as white residents departed from the area for a variety of reasons. Falling property prices, a by-product of capital flight after the Soweto Uprising of 1976, made it possible for many white tenants in Hillbrow to move out and buy houses in the suburbs. At the same time, young white adults were electing to stay with parents rather than moving into flats of their own, as the cost of living surged following the oil crisis of the late 1970s. Finally, the pool of young white men needing accommodation declined following the extension of compulsory national military service to two years in 1977. As a consequence of these factors, many flats in Hillbrow were left empty [21].

The early 1980s saw a number of important political shifts. The increasingly reformist Nationalist Party (NP) government began to court Indians and Coloureds, promising these constituencies limited political power – a promise that was partly realised in the formation of a ‘Tricameral Parliament’ in 1983. Lacking the financial capacity and political will to build new Indian and Coloured suburbs, however, the NP-controlled management committee of the Johannesburg City Council recommended in 1982 that Hillbrow should be declared a ‘mixed’ area to allow Indians and Coloureds to live there. These developments contributed to the failure – or, perhaps, the reluctance – of the government to check a steady influx of Indian and Coloured families into Hillbrow in the early 1980s. By 1985, an estimated 70% of apartments were occupied by whites, 25% by coloureds and Indians, and 5% by blacks [17].

In 1986, after three years of constant protests in black townships across South Africa, the worst recession since the 1930s and growing international pressure on the apartheid state, the NP government scrapped the Pass Laws and began to relax the monitoring of the Group Areas Act for blacks. As more black residents moved into Hillbrow, white residents steadily began to move out – concerned about the crime and growing deterioration of parts of the suburb, which they blamed on its changing racial composition. An estimated 25 to 30% of flats in the Hillbrow-Joubert Park-Berea area were vacant by 1987 [4]. The combination of supply and demand, together with the legislation changes cited above, meant that during 1987 some blocks of ‘all-white’ flats in Hillbrow rapidly transformed into ‘all-black’ flats in just a few months [17].

By the early 1990s, black South Africans “had appropriated the neighbourhood numerically and psychologically” [4]. This shift applied not only to residential spaces in Hillbrow but also to entertainment venues and schools. The few remaining clubs and hangouts were sold to new owners. For example, the Chelsea Hotel, a popular white hangout, was re-orientated to accommodate black patrons, but went into liquidation in 1998. The Johannesburg School for Girls became non-racial in 1990 and the following year it had 400 black pupils and just five white pupils, a ‘racial tipping’ process of astonishing rapidity. Nonetheless, Hillbrow remained ‘multiracial’ as a whole and by 1990, white residents still made up about 20% of its population [22].

The process of Hillbrow’s desegregation was not without struggle. The neo-fascist British National Front had offices in South Africa and in the late 1970s, its members led searches in Hillbrow for non-white tenants to evict them from their homes [21]. In June 1989, following the scrapping of the Separate Amenities Act, a group of black people came to swim in the Hillbrow public pool as a celebration of the end of Apartheid legislation relating to public amenities. This provoked a response from the right wing organisation, the *Afrikaans Weerstandsbeweging* (Afrikaner Resistance Movement), whose members attempted to stop the swimmers and distributed racist literature [23].

Civil movements played an important role in supporting residents’ rights. Organisations such as the Action Committee to Stop Evictions (Actstop) in 1979 supported residents whose tenancy was threatened, mainly through challenges mounted by volunteers from the legal fraternity [21]. This culminated in the landmark court case, *Govender* versus *the State* [24], in which Judge Goldstone concluded that convictions under the Group Areas Act were unjust and had to be halted [21]. By the 1990s, there was minimal racist hostility in Hillbrow, although by this time most conservative whites had left the suburb. According to surveys undertaken at the time, over 80% of residents thought that racial barriers had broken down; most “were delighted” with this development and only 5% thought that racial tension still existed in their apartment buildings [4].

In this era, Hillbrow emerged as an island of tolerance and non-racialism. In part, the suburb’s pioneering and progressive spirit may have been influenced by its youthful population: even by the early 1990s, 55% of the population were under 30 years of age and 80% were under 40 years [4]. Historians have also observed that the changing racial attitudes in Hillbrow can largely be attributed to gay activism in the area in the late 1970s and early 1980s. Gevisser and Cameron note: “As Hillbrow became a grey area in later years – a white neighbourhood where black people could live, illegal and unprosecuted, in relative safety - it became ‘pink’ in the 1970s: a heterosexual neighbourhood where gay people could live in relative safety” [25]. It was the presence of gay people living in Hillbrow that turned the area into a tolerant, “liberated zone” of sorts, laying the ground for it to become Johannesburg’s first deracialised neighbourhood in the 1980s. It was a place where “those seeking to identify with subcultures relatively removed from the moral confines of suburban white South Africa” could feel safe [19]. Yet, by the late 1990s, when most of the white residents had moved out, Hillbrow appeared to have abandoned this character.

## Hillbrow’s Decline: 1994–2002

*“... the owners of this building they don't care especially where there are black people. They don't paint, maintenance is poor. ... They just want to collect money. They don't care about the welfare of the people (Hillbrow Resident).”* [26].

The mid to late 1990s represented an era of severe decline: buildings became increasingly decrepit, some lacked basic services such as electricity, sewerage and water, and there was a noticeable trend toward overcrowding. Both white and middle class black residents had become increasingly disenchanted by the increasing grime and crime and gradually left the neighbourhood. Morris sums this up: “…by the late 1990s, the persistence of decline, crime and racism, ensured that the racial diversity that had pertained in the early part of the 1990s was history” [4]. Fears about petty and violent crime contributed toward this trend. In 1997, police statistics recorded 96 murders, 576 robberies, 48 cases of housebreaking, 324 vehicle break-ins, and 504 thefts of vehicles. About half of all crimes in the precinct served by the Hillbrow Police Station took place in Hillbrow itself, especially drug-related illicit activity. Mugging was a serious problem, with one in five residents having been mugged and one in ten mugged more than once. Between 1996 and 2000, armed robbery almost doubled, as did assaults [27]. Not surprisingly, this led to residents remaining in their apartments where they felt safe, and few ventured out at night [4]. Other aspects of living in a congested urban setting contributed toward resident’s growing dissatisfaction with the area, including the incessant noise from taxis, music from nightclubs and domestic violence among neighbours [4].

The decline of inner-city neighbourhoods such as Hillbrow is often attributed to racial desegregation [28]. Yet in this case, urban decline had more to do with the dynamics of landlord-tenant relationships that resulted in overcrowding and the physical deterioration of buildings, than with the shifting racial profile of the area [17, 21]. Moreover, the deterioration of Hillbrow arguably has its origins in the early 1970s, preceding the era of desegregation.

A survey conducted in 1991 in the inner-city revealed a direct correlation between overcrowding of apartments (up to 24% were defined as overcrowded) and a lack of services. Those buildings with more than 5 occupants per bedroom, for example, experienced an almost permanent lack of electricity, irregular supplies of water and drainage and sewerage systems. This was primarily due to owners and landlords withdrawing services and to the absence of caretakers. Eager to fill their properties with new residents who would not be eligible for rent control, landlords encouraged overcrowding [28]. In some cases, however, buildings were deliberately destroyed by tenants as political acts. In the mid to late 1990s, black tenants in some buildings purposefully contributed toward their decline as an act of defiance against the apartheid state. Perceiving landlords as the beneficiaries of apartheid, they cast acts of vandalism and refusal to pay for services as acts of resistance [29].

In the late 1990s and early 2000s, South Africa experienced an influx of foreign nationals, and Hillbrow continued to be the main port of entry for refugees and economic migrants [30]. In his survey conducted in 1997, Morris found that 87% of residents were South African born, and the remaining 13% came from Europe (5%), Southern Africa (7%) and elsewhere in Africa (1%) [4]. Just a few years later, a different picture emerged: in a survey of residential hotels in Hillbrow undertaken in 2002, between 25 and 38% of residents were foreign born, while 89% had arrived in South Africa in the past 5 years or less [27]. Similarly, another study has estimated that 90% of the inner-city’s total resident population arrived there only after the early 1990s [30].

Hillbrow’s shifting demographics – the new wave of foreign nationals and refugees in particular – have led to increasing levels of xenophobia and violence. Nearly two thirds of the non-South Africans interviewed in a survey of 200 residents of hotels in Hillbrow in 2002 reported that they feared assault by South Africans because of their foreign status [31]. Sadly, the fears of foreign residents were realised all too often in the years that followed: Hillbrow was among the areas targeted in the xenophobic violence that swept through Gauteng in 2008, leaving shops looted, homes destroyed, and more than 60 people dead [32, 33].

## Early responses to HIV in Hillbrow: 1980–2016

*“…a young man who died of a strange illness (…). The migrants said it could only have been AIDS. After all, was he not often seen roaming the whorehouses and dingy pubs of Hillbrow (…) But strange illnesses courted in Hillbrow, (…) could only translate into AIDS.”* [2]

Having sketched a social, political and economic history, what remains to be explored is the impact of the HIV epidemic on the people of Hillbrow and the nature of the public health response it generated. In the public imagination, Hillbrow’s long association with sex work, criminality and foreigners, has provided fertile ground for constructions of the suburb as “emblematic of diseases of the social body, an ambiguous space of potential infection and contamination” [Nuttall and Mbembe cited in [34]]. It is therefore appropriate that Hillbrow became the setting for some of the earliest HIV prevention and support centres in the city.

Mirroring global trends, in the early years of the epidemic in Johannesburg, HIV largely affected white gay men. Despite the relatively low numbers of infections, the media hysterically coined AIDS a ‘gay plague’. Yet, by 1990 the epidemic amongst homosexuals was already ‘levelling off’ and a decade later only 207 homosexual men were reported to be infected with AIDS in South Africa [35]. While the country as a whole was slow to respond to the epidemic, one of the first centres of HIV care and prevention was launched in Hillbrow, in the form of the AIDS Training Information Centres (ATIC), established at the Esselen Street Clinic in 1990. At this time, very few services existed for gay men living with HIV, and the Hillbrow ATIC provided much-needed counselling services, testing and education. It ran group psychotherapeutic sessions that focussed on preparing for death. Importantly, the centre challenged conventional approaches to HIV prevention, which at the time was based on abstinence, faithfulness, and condom use, or “ABC”. It also defied South African censorship laws and the recently abolished laws preventing interracial relationships. Yet, by the late 1990s, as the epidemic shifted, so did the clientele of the ATIC change from mostly white gay men to predominantly black heterosexual women [36].

More broadly, attention was turning to the provision of health services for the large sex worker population in Hillbrow, many of whom were at risk of HIV infection. While in the mid-1980s HIV infection was virtually absent in this population [37], by 1997, 45% of 247 sex workers surveyed tested positive for HIV [38]. The total population of sex workers in the Johannesburg inner-city is hard to assess, but has been estimated at between 5000 and 10,000 [39]. Many women who defined themselves as sex workers avoided seeking care in government clinics owing to stigma and discrimination, resulting in untreated STI and other health issues [5]. In 1994, the Reproductive Health Research Unit (now the Wits Reproductive Health and HIV Institute, or WRHI) of the University of the Witwatersrand initiated Thursday evening health services for sex workers in Hillbrow, in addition to existing treatment for sexually transmitted infections (STI) at the Esselen Street Clinic. By 1998, services were expanded to include taking mobile clinics into local brothels to meet the growing need [39]. In around half of the identified brothels, these mobile clinics offered sex workers clinical services including treatment of symptomatic STIs, health education, safe-sex negotiation skills, and condoms [40]. In addition, outreach workers visited the numerous bar lounges and beer halls in Hillbrow to engage with men [39]. An assessment of the mobile services concluded that these efforts had transformed the perception of brothels from “diseased and dirty” to “safe and healthy” [5].

By the early-to mid-2000s, South African HIV infection rates had reached catastrophic levels, followed by dramatic increases in morbidity and mortality. Treatment for HIV infection was limited to containing opportunistic infections, and palliative care – until universal access to ARVs was endorsed by the Constitutional Court in 2002 [41]. In 2003, the Hillbrow Health Precinct became home to one of the largest HIV treatment sites in South Africa, in a PEPFAR-funded collaboration between the WRHI, the City of Johannesburg, and the Department of Health. This site, located in the former Johannesburg Hospital building, has more than 17,000 patients on treatment.

## Conclusion

Originally envisaged as the ‘healthiest’ of suburbs, this high-rise inner-city suburb situated in the clean air above the sprawl of mining camps in old Johannesburg later became a progressive space of racial integration and experimentation with newfound identities. Although its more recent history has been marked by corruption, crime, poverty and decline, and while longstanding associations with sex work and drug use persist, a more positive reading of the last decade may yet be possible. Various examples of neighbourhood development have emerged in Hillbrow in recent years. These include efforts such as The Better Buildings Programme through the Johannesburg Property Company that intends to reclaim and rehabilitate buildings taken over by slumlords, and the EKhaya Neighbourhood Programme that seeks to clean up neighbourhoods and make the streets safer [42] .

Such initiatives hint at the existence of a more optimistic narrative that highlights urban renewal, health and social justice. The neighbourhood may host a highly transient population and display radically shifting demographics, but the one constant that endures across Hillbrow’s 120 year long history is the concentration of cutting-edge public health interventions within its boundaries. Together with the high quality health services and multidisciplinary research unfolding within the confines of the Hillbrow Health Precinct, there is much that is encouraging about Hillbrow’s future.

## Abbreviations

ABC: Abstain, Be faithful, condomise
Actstop: Action committee to stop evictions
AIDS: Acquired immunodeficiency syndrome
ATIC: AIDS Training and Information Centre
ANC: African National Congress
PEPFAR: President’s Plan for AIDS Relief
HIV: Human Immunodeficiency Virus
NP: Nationalist party
RHRU: Reproductive health research unit
STI: Sexually transmitted infections
TB: Tuberculosis
Wits RHI: Wits reproductive health and HIV institute

## Footnotes

1 Racial categories (‘black’, ‘white’, ‘Indian’, and ‘coloured’) are used consistently throughout the paper. This does not reflect the authors’ endorsement of the former apartheid government’s use of racial categories and population groups.

2 This is most evident in the internet blogs and commentaries by largely white South Africans on the decline of the inner city. For example, see https://johannesburg1912.wordpress.com/2016/02/09/history-of-hillbrow-pt-2/

3 The 1913 Land Act aimed at regulating the acquisition of land, placed under communal tenure and controlled by chiefs. Land could neither be sold nor purchased, and black tenant farming was forbidden. This effectively forced large numbers of black men into wage labour.

4 Passed by the Apartheid government, the Group Areas Act enforced the segregation of different races, categorizing specific urban neighbourhoods or zones according to race, and restricting ownership and the occupation of land in these zones to a specific statutory (racial) group.

5 The Pass Laws Act of 1952 made it compulsory for all black South Africans over the age of 16 to carry the “pass book” at all times.
